# Molecular Epidemiology of New Delhi Metallo-*β*-Lactamase-Producing *Escherichia coli* in Food-Producing Animals in China

**DOI:** 10.3389/fmicb.2022.912260

**Published:** 2022-07-01

**Authors:** Xu Kuang, Yan Zhang, Juan Liu, Run-Shi Yang, Zhi-Ying Qiu, Jian Sun, Xiao-Ping Liao, Ya-Hong Liu, Yang Yu

**Affiliations:** ^1^National Risk Assessment Laboratory for Antimicrobial Resistance of Animal Original Bacteria, South China Agricultural University, Guangzhou, China; ^2^Guangdong Provincial Key Laboratory of Veterinary Pharmaceutics Development and Safety Evaluation, South China Agricultural University, Guangzhou, China; ^3^National Reference Laboratory of Veterinary Drug Residues, College of Veterinary Medicine, South China Agricultural University, Guangzhou, China; ^4^Guangdong Provincial Key Laboratory of Microbial Safety and Health, State Key Laboratory of Applied Microbiology Southern China, Institute of Microbiology, Guangdong Academy of Sciences, Guangzhou, China; ^5^Guangdong Laboratory for Lingnan Modern Agriculture, Guangzhou, China

**Keywords:** NDM, food-producing animals, epidemiology, antimicrobial resistance, the environment

## Abstract

We conducted a molecular surveillance study for carbapenem-resistant *Enterobacteriaceae* (CRE) colonization in food-producing animals in China that included primarily swine and poultry for three consecutive years. A total of 2,771 samples from food-producing animals and their surrounding environments were collected from different regions in China from 2015 to 2017. Enrichment cultures supplemented with meropenem were used to isolate carbapenem non-susceptible isolates and these were subsequently identified by MALDI-TOF MS. Resistance phenotypes and genotypes were confirmed using antimicrobial susceptibility testing and molecular biological techniques. Genomic characteristics of the carbapenemase-producing isolates were investigated using whole genome sequencing (WGS) and bioinformatic analysis. In total, 88 NDM-positive *Enterobacteriaceae* were identified from 2,771 samples and 96.6% were *Escherichia coli*. The New Delhi metallo-*β*-lactamase (NDM)-positive *E. coli* displayed a diversity of sequence types (ST), and ST48 and ST165 were the most prevalent. Three variants of *bla*_NDM_ (*bla*_NDM-1_, *bla*_NDM-4_, and *bla*_NDM-5_) were detected and WGS indicated that *bla*_NDM-5_ predominated and was carried primarily on IncX3 plasmids. All these isolates were also multiply-drug resistant. These results revealed that food-producing animals in China are an important reservoir for NDM-positive *E. coli* and pose a potential threat to public health.

## Introduction

Antimicrobial resistance (AMR) is rising to high levels in both human and veterinary health. New threats arising from multidrug-resistant bacteria and the very limited number of antibiotics coming to the market and bring us perilously to the end of antibiotic era where common treatable infections can once again be fatal. The abuse of human antibiotics in food-producing animals as prophylactic agents or growth promoters has become an important contributing factor to increasing AMR (Van et al., 2020). Food-producing animals could serve as reservoir for antibiotic-resistant bacteria that can be transmitted to humans through direct contact or the food chain (Founou et al., 2016; Wang et al., 2017). Therefore, the prevalence of AMR will continue to rise worldwide and become one of the most serious threats to human and animal health.

Among the major multidrug-resistant bacteria, the emergence and dissemination of carbapenem-resistant *Enterobacteriaceae* (CRE) is identified as one of the most serious threats to human and animal health worldwide (Mariana Huband et al., 2017; Zhang et al., 2017, 2018; Bonomo et al., 2018). Notably, the recent increase in CRE was primarily because of the acquisition of carbapenem hydrolyzing enzymes such as the New Delhi metallo-*β*-lactamase (NDM; Kumarasamy et al., 2010; Munoz-Price et al., 2013). Since the first report of NDM-1 in a *Klebsiella pneumoniae* isolated from a patient in 2009, NDM-positive *Enterobacteriaceae* have been rapidly spreading in hospitals as well as in food-producing animals around the world and especially in China (Yong et al., 2009; Logan and Weinstein, 2017). To date, NDM-positive *Enterobacteriaceae* have been found on swine, geese farms, cow and chicken in China (Kong et al., 2017a; Köck et al., 2018). The spread of NDM-positive *Escherichia coli* has been documented among chickens, swallows, farmers, dogs and even flies in a chicken farm (Berrazeg et al., 2014; Logan and Weinstein, 2017; Li et al., 2019).

However, the dissemination of NDM-positive *E. coli* among food-producing animals in China is currently poorly understood. A large-scale survey of the NDM-positive *Enterobacteriaceae* covering a large region of China has yet to be performed. In this study, we examined the long-term and large-scale prevalence and molecular epidemiological features of NDM-positive *E. coli* from livestock animals from northern to southern China for three consecutive years, 2015–2017.

## Materials and Methods

### Sample Collection and Bacterial Isolation

**Figure 1:**
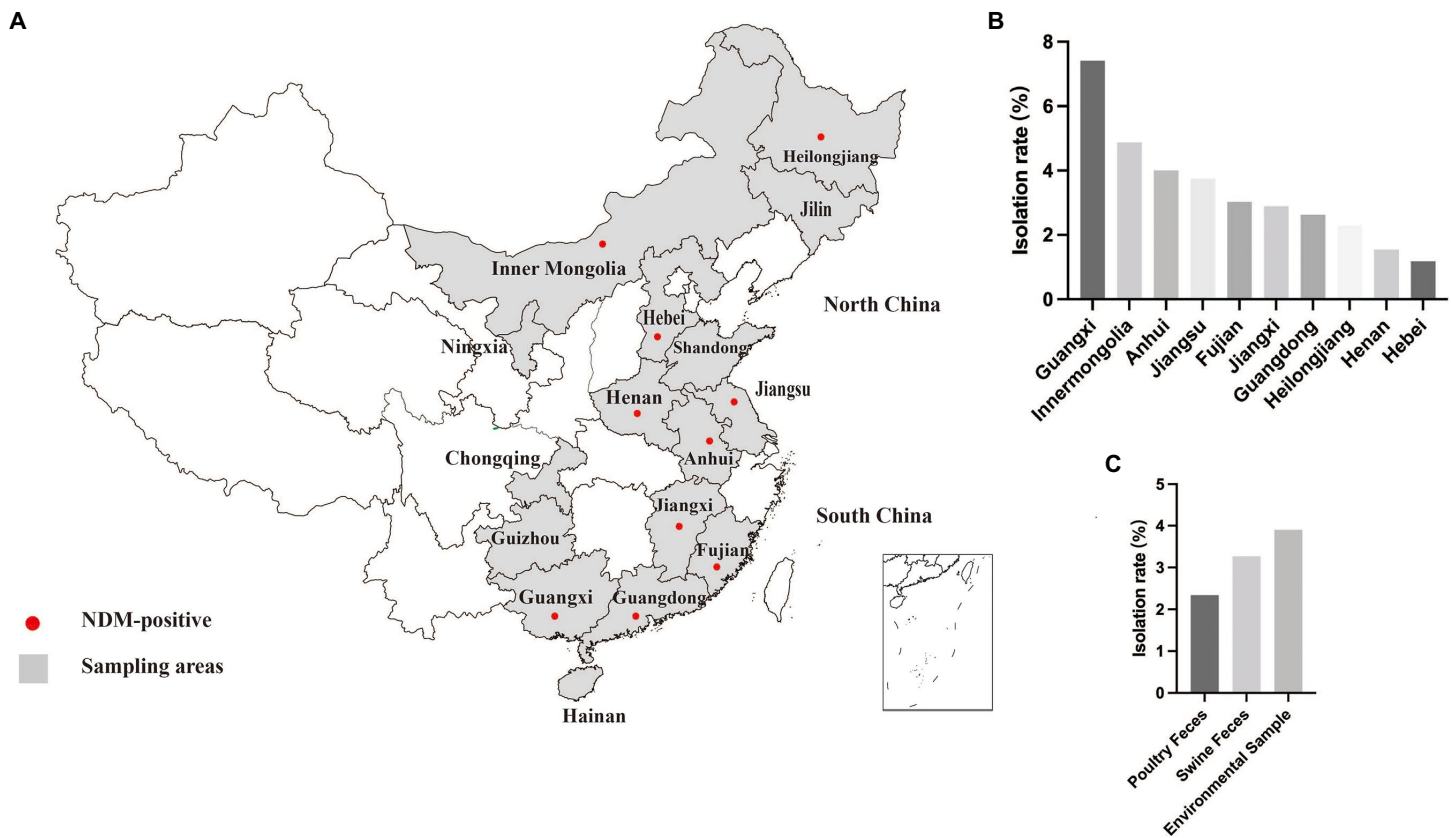
Sampling areas and detection rate. **(A)** Sampling areas for poultry and swine farms in China examined in this study. **(B)** Detection rate of *bla*_NDM_-positive isolates in different provinces. **(C)** Detection rate of *bla*_NDM_-positive isolates in different type of samples.

A total of 2,771 samples, including 526 samples in 2015, 812 samples in 2016, and 1,433 samples in 2017 were collected from food-producing animals (fecal samples) on poultry and swine farms and their surrounding environments (Supplementary Table S1). Sixteen provinces that play important roles in the swine and poultry industry from north to south China were randomly selected for sampling. According to the north–south demarcation zone, eight provinces that include Jiangsu, Henan, Heilongjiang, Hebei, Jilin, Ningxia, Jiangsu, Inner Mongolia are situated in the north China while the remainder were located in south China (Figure 1).

Environmental samples (sewage and soil) were incubated overnight in Luria Bertani broth without antibiotics. Environmental cultures and diluted feces samples were directly plated on MacConkey agar with 1 mg/l meropenem and incubated at 37°C. Carbapenem non-susceptible isolates were identified using MALDI-TOF MS (Shimadzu Biotech) and 16S rDNA sequencing (Seng et al., 2009; Walsh and Duffy, 2013) Carbapenemase production was examined for all isolates using the Carbapenemase Nordmann-Poirel test (Nordmann et al., 2012b). All the carbapenemase-producing isolates were screened using polymerase chain reaction (PCR) for the presence of *bla*_NDM_, *bla*_OXA-48-like_, *bla*_VIM_, *bla*_KPC_, and *bla*_IMP_ (Poirel et al., 2011). In addition, the entire *bla*_NDM_ amplicons were sequenced (Sanger) and the 813 bp ORF of *bla*_NDM_ was typed using BLAST.^1^

### Antimicrobial Susceptibility Testing

The minimal inhibitory concentrations (MIC) of 14 antibiotics (amikacin, gentamicin, meropenem, imipenem, ertapenem, cefoxitin, cefotaxime, ceftazidime, aztreonam, cefoxitin, tetracycline, fosfomycin, trimethoprim/sulfamethoxazole, and florfenicol) for the isolates were determined by agar dilution and interpreted according to the clinical and laboratory standard institute (CLSI) documents M100-S28 and VET01-S2. The breakpoints of tigecycline and colistin for *Enterobacteriaceae* were interpreted according to the EUCAST criteria (Version 6.0). *E. coli* American Type Culture Collection (ATCC) 25922 served as a quality control strain for the testing.

### Pulsed-Field Gel Electrophoresis (PFGE) Typing, Whole-Genome Sequencing and Conjugation Assay

The genetic relatedness of NDM-positive isolates was investigated using *Xba*I-PFGE for *E. coli*. PFGE patterns were analyzed with the Dice coefficient and the unweighted pair group with arithmetic mean clustering method using BioNumerics (Applied Maths, Ghent, Belgium), resulting in PFGE patterns with > 90% similarity between clusters. Based on different resistance phenotypes and genetic relationships, 76 strains were selected for Whole-Genome Sequencing (WGS). Genomic DNA was extracted from isolates using the Genomic DNA Purification Kit (Tiangen, Beijing, China). WGS was performed with the Illumina HiSeq 2,500 system (Novogene Guangzhou, China). Gene annotation and prediction were performed using RAST^2^ and BLAST. MLST sequence types (ST), plasmid incompatibility (Inc) groups and antibiotic resistance genes (ARGs) were analyzed using software from IS finder^3^ and the Center for Genomic Epidemiology.^4^

Streptomycin-resistant *E. coli* strain C600 was used as the recipient to determine the transferability of the resistance genes. The conjugation assays were performed using the filter mating method and the proportion of donor to recipient bacteria stands 1:1. Transconjugants were selected using MacConkey agar plates containing both 2,000 mg/l streptomycin and 1.0 mg/l meropenem. Selected transconjugants were confirmed by PCR.

## Results

### Bacterial Isolation and Detection of Carbapenemase Genes

In this study, 654 carbapenem non-susceptible bacteria (≥ 1 mg/l) were recovered from 2,771 fecal and environmental samples that included sewage and soil samples from 16 provinces in China from 2015 to 2017. We identified the presence of the *bla*_NDM_ gene in 88 isolates. In 2015, the prevalence of NDM-positive *Enterobacteriaceae* detected in samples was 1.52% (8/526) and the positive samples were primarily from Jiangsu and Guangdong. The prevalence for 2016 was almost identical (1.48%, 12/812). Interestingly, the prevalence increased dramatically to 4.82% (69/1433) in 2017 (Supplementary Table S1). Among all the provinces, *bla*_NDM_ was the most prevalent in Guangxi. Notably, samples collected from six provinces, including Jilin, Shandong, Ningxia, Chongqing, Guizhou and Hainan did not detected NDM-positive strain (Figure 1). Species identification and WGS analysis indicated that the 88 *bla*_NDM_-carrying isolates belonged to three *Enterobacteriaceae* species, including *E. coli* (*n* = 82), *K. pneumoniae* (*n* = 5) and *Citrobacter freundii* (*n* = 1).

### Antimicrobial Susceptibility Testing and Conjugation Assays

Our collection of 88 NDM-positive isolates were found to be resistant to imipenem (MIC 4 to > 64 mg/l), meropenem (MIC 4 to > 64 mg/l) and ertapenem (MIC 16 to > 64 mg/l) as well as concurrently resistant to the other *β*-lactams cefotaxime, cefoxitin and ceftazidime. Moreover, most were also resistant to trimethoprim/sulfanilamide (96.6%; *n* = 85; MIC 40 to > 320 mg/l), tetracycline (94.3%; *n* = 83; MIC 16 to > 256 mg/l), florfenicol (80.6%; *n* = 71; MIC 16 to > 256 mg/l), ciprofloxacin (72.7%; *n* = 60; MIC 4 to > 256 mg/l), and gentamicin (59.1%; *n* = 52; MIC 16 to > 256 mg/l). However, we found relatively a lower prevalence for resistance to aztreonam (45.5%), amikacin (34.1%), Fosfomycin (29.5%), colistin (28.4%) and tigecycline (11.4%). These results showed that NDM-positive isolates displayed reduced susceptibilities to β-lactam antibiotics, but remained susceptible to colistin and tigecycline. Conjugation experiments using these isolates resulted in the transfer of *bla*_NDM_-carrying plasmids from 76 isolates (86.5%) to *E. coli* C600str recipient (Supplementary Table S2).

### Phylogenetic Analysis of NDM-Positive *Escherichia coli*

PFGE was successfully performed on these 77 *bla*_NDM_-carrying *E. coli* and resulted in 74 PFGE patterns. There were four groups of isolates that shared similar PFGE patterns including FS21-4 and FSCK25-1, HB14-1 and HB14-3, JZ33 and JZ41-2; most isolates of each PFGE group were from the same area, while HN68-2 and FCG47-1 were from different provinces and years but shared > 90% similarity in PFGE patterns. The remaining 69 isolates belonged to different phylogenetic groups (Supplementary Figure S1). Based on PFGE profiles, a total of 72 isolates were selected for WGS and this analysis demonstrated that these *E. coli* isolates had 38 distinct STs. The most prevalent STs were ST48 (10/72; 13.9%), ST165 (6/72; 8.3%) and ST405 (4/72; 5.6%).

### Resistance Profiles

**Figure 2:**
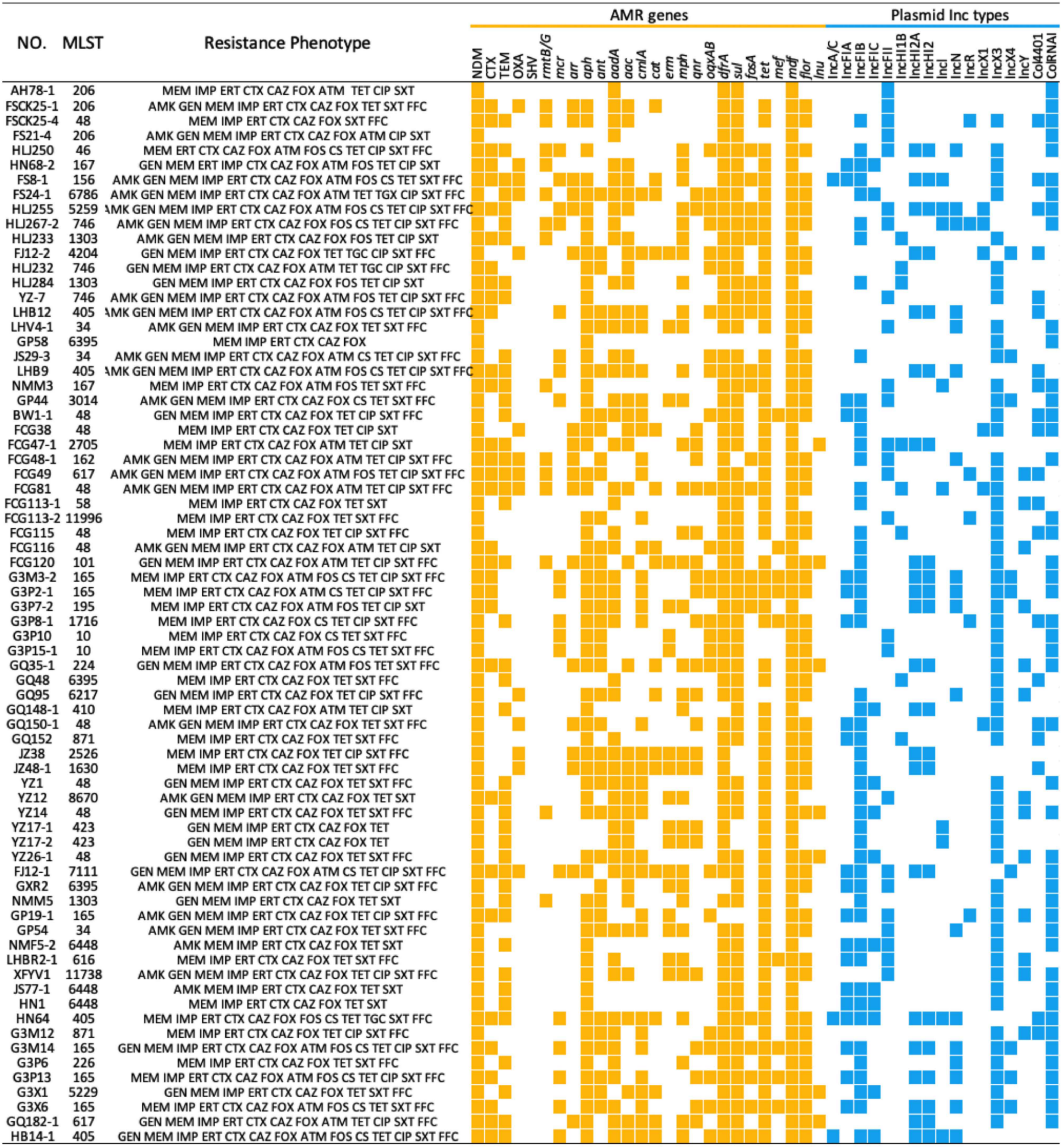
MLST, Resistance Phenotype, ARG and plasmid replicons of 72 NDM-positive *Escherichia coli* isolates from food-producing animals in China. The heatmap was generated after aligning the contigs of sequenced genomes of each strain to MLST, Resfinder and PlasmidFinder. ARGs and plasmid Inc. types are indicated by yellow and blue squares, respectively. AMK, amikacin; GEN, gentamicin; MEM, meropenem; IMP, imipenem; ERT, ertapenem; CTX, cefotaxime; CAZ, ceftazidime; FOX, cefoxitin; ATM, aztreonam; FOS, fosfomycin; CS, colistin; TET, tetracycline; TGC, tigecycline; CIP, ciprofloxacin; SXT, sulfamethoxazole-trimethoprim. FFC, florfenicol.

Whole genome sequencing analysis revealed that NDM-positive *E. coli* isolates possessed > 30 distinct ARGs conferring resistance to nearly all currently available antibiotics. Among the other 24 ARG types, 11 ARGs were common with a prevalence > 50% and these included *bla*_TEM_ (*n* = 43), *aph* (*n* = 64), *ant* (*n* = 36), *aadA* (*n* = 49), *aac* (*n* = 40), *cmlA* (*n* = 37), *dfrA* (*n* = 68), *sul* (*n* = 65), *tet* (*n* = 65), *mdf* (*n* = 72), and *flor* (*n* = 53). These genes conferred resistance to seven classes of antibiotics; β-lactams, sulphonamides, quinolones, tetracyclines, chloramphenicol, aminoglycosides and macrolides. Notably, gene *mcr-1* mediating resistance to the last-resort antibiotic colistin (Poirel et al., 2017; Li et al., 2020) was detected in 19 NDM-positive *E. coli* isolates. Additional analysis of plasmid sequences indicated that 72 NDM-positive *E. coli* isolates in this study carried 14 types of Inc. plasmids. IncX3 predominated and IncFIB (65.3%, 47/72) and IncFII (34.7%, 25/72) were also highly represented (Figure 2).

### Genetic Environments of *bla*_NDM_

**Figure 3:**
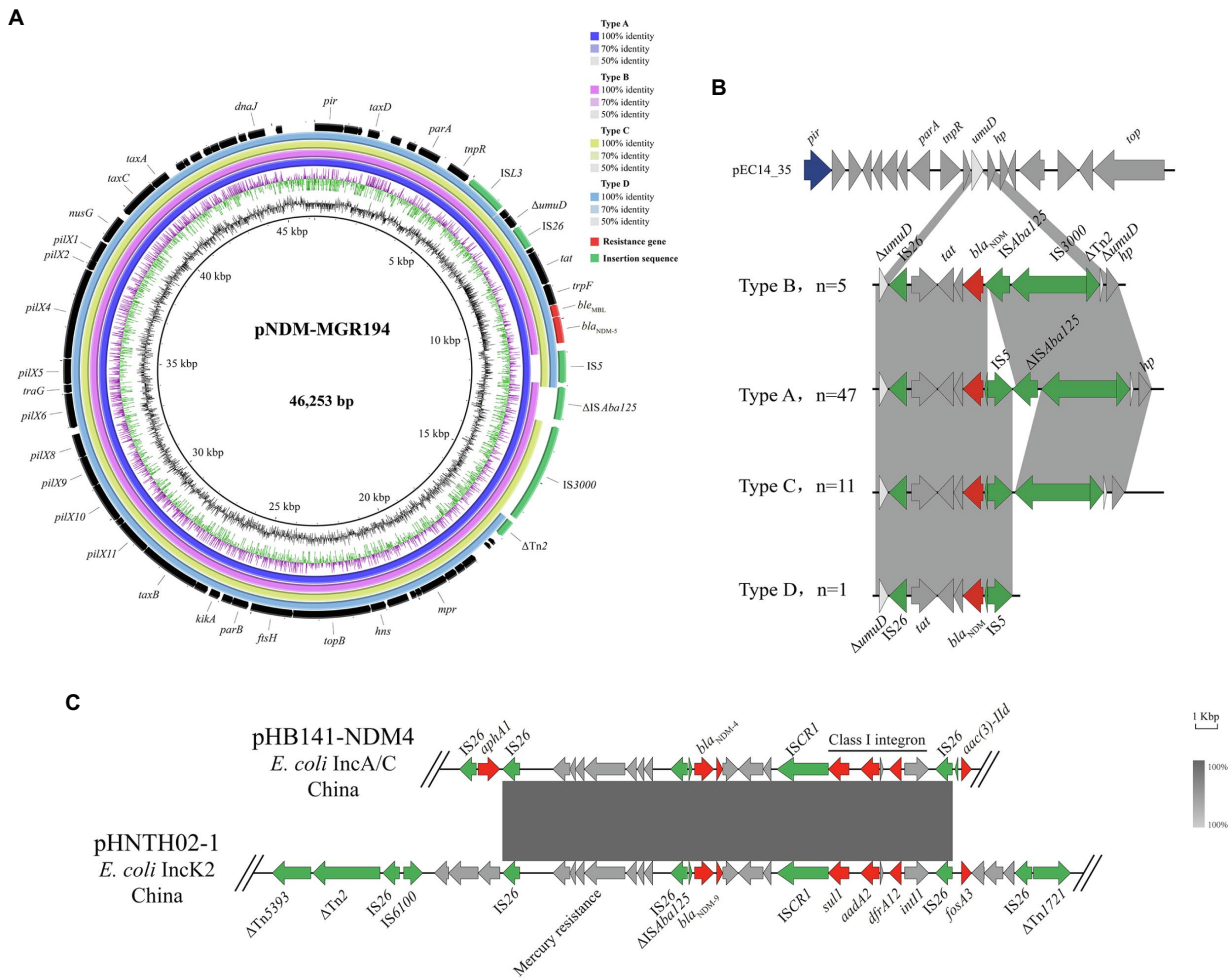
Genetic environments for *bla*_NDM_. **(A)** Comparison of *bla*_NDM-5_-carrying IncX3 plasmids with reference sequence pNDM-MGR194. **(B)** Four different genetic environments on the IncX3 plasmid harboring *bla*_NDM._ **(C)** Linear sequence comparison of pHB141-NDM4 and pHNTH02-1.

A sequence comparison analysis revealed that all 64 IncX3 plasmids were similar to a *bla*_NDM-5_-carrying IncX3 plasmid pNDM-MGR194 (Acc. No. NC_022740) from a *K. pneumoniae* isolate. These 64 plasmids shared an identical and conserved IncX3 backbone, and their inserted *bla*_NDM_ sites were all near the *umuD* gene. Differences between these plasmids were primarily contained within the *bla*_NDM-5_ region between the *par*A and IS*3000* genes. The diversity within this region was used to separate these 64 plasmids into four groups (Types A–D). Type A plasmids (*n* = 47) shared the highest similarity with the reference plasmid. Types B (*n* = 5), C (*n* = 11), and D (*n* = 1) omitted IS*5*, ΔISA*ba125* and ΔISA*ba125*-IS*3000*-ΔTn*2*, respectively (Figure 3A). In the Type B group, *bla*_NDM_ was located in the structure IS*26*-*tat*-*trp*F-*ble*_MBL_-*bla*_NDM_-IS*A*ba*125*-IS*3000*-ΔTn*2*. The structure was inserted into the plasmid pEC14-35 (Acc. No. NC_019083) and therefore formed the genetic environments of *bla*_NDM_ for Type B. Type A most likely was formed by IS*5* insertion into the region between *bla*_NDM_ and IS*A*ba*125*. Types C and D emerged after losing IS*A*ba*125* and IS*A*ba*125*-IS*3000*-ΔTn*2* most likely due to homologous recombination (Figure 3B).

In addition, the genetic environments of a *bla*_NDM-4_-bearing IncA/C plasmid (pHB141-NDM4) demonstrated that the ARGs *sul1*, *aadA2* and *dfrA12* were packaged in Class I integrons located within the structure of IS*Aba125*-*bla*NDM-4-*ble*MBL-*trpF*-*tat*-Δ*cutA*. There were also a series of genes mediating Hg resistance downstream of *bla*_NDM-4_. Two IS*26* elements flanking the *aphA1* gene were also downstream of *bla*_NDM-4_. A sequence comparison analysis revealed that all the 64 IncX3 plasmids were similar to a *bla*_NDM-9_-carrying IncK2 plasmid pHNTH02-1 from *E. coli* in China (Acc. No. NZ_MG196294). The reference sequence featured a composite transferable structure of IS*26*-ΔIS*Aba125*-*bla*NDM-*ble*MBL-*trpF*-*tat*-Δ*cutA*-IS*CR1*-*sul1*-*aad2*-*gcuF*-*dfrA12*-*intI1*-IS*26*, with two inverted repeat sequences ΔTn*1721* and ΔTn*5393* (Figure 3C).

## Discussion

The presence of NDM-positive *Enterobacteriaceae* in livestock animals is of concern because this may facilitate expansion of the gene pool from which pathogenic bacteria can acquire ARGs and consumers may subsequently be exposed *via* the food chain (Woodford et al., 2014; Mollenkopf et al., 2017). For this reason, we conducted a long-term and large-scale characterization of the prevalence and genetic relationships between NDM-positive bacteria from livestock animals in China from 2015 to 2017. Since its first report in British, *bla*_NDM-5_ has been detected in clinical isolates from Europe, Asia, South America, the United States of America and Australia (Nakano et al., 2014; Wailan et al., 2015; Baek et al., 2019; López-Hernández et al., 2020; Ramadan et al., 2020; Costa et al., 2021). Notably, the prevalence of *bla*_NDM-5_ isolates from food-producing animals has been sporadic in aboard (Ghatak et al., 2013), although there are more relevant reports in China (Yang et al., 2016; Kong et al., 2017a; Yao et al., 2020; Zhao et al., 2021; Lv et al., 2022). In the current study, our results revealed a wide contamination of NDM-positive *Enterobacteriaceae* in ten different provinces. In particular, the prevalence of *bla*_NDM_-carrying *Enterobacteriaceae* in swine and poultry in Guangxi province (10.3%, 32/312) was much higher than for 2017 and suggested that food-producing animals in Guangxi province have been severely contaminated by carbapenem-resistant *Enterobacteriaceae*. Fortunately, three northern provinces Jilin, Ningxia and Shandong, and three southern provinces Chongqing, Guizhou and Hainan had not been contaminated by NDM-positive *Enterobacteriaceae* over our 3-year study period.

While carbapenem use is prohibited in swine and poultry production chains, different *Enterobacteriaceae* species and various *bla*_NDM_ variants have been found on livestock farms (Zhai et al., 2020; Wang et al., 2021). In the current study, we identified 88 NDM-positive isolates belonging to three *Enterobacteriaceae* species including *E. coli*, *K. pneumoniae* and *C. freundii*. For the NDM-positive *E. coli* isolates, the predominant variant of *bla*_NDM_ was *bla*_NDM-5_. There are to date seven *bla*_NDM_ variants (*bla*_NDM-1_, *bla*_NDM-4_, *bla*_NDM-5_, *bla*_NDM-7_, *bla*_NDM-9_, *bla*_NDM-17_, and *bla*_NDM-20_) that have been detected in food-producing animals in China and *bla*_NDM-5_ was the most prevalent and was found in poultry, swine, geese and ducks (Liu et al., 2017, 2018; Pruthvishree et al., 2017; Cen et al., 2021; Wang et al., 2021). Furthermore, our PFGE analysis illustrated that the genetic background of NDM-positive *E. coli* varied considerably across the country although there were a small number of closely related strains in the same regions. Therefore, clonal spread was not the primary mode of *bla*_NDM_ gene transmission and was consistent with the results of previous studies (Mantilla-Calderon et al., 2016; Zhang et al., 2018). Our WGS results also demonstrated that these *E. coli* isolates had 38 distinct STs and could be distinguished by their geographical distribution. For instance, ST48, ST165 and ST405 were more prevalent than the others. ST48 *E. coli* isolates have been linked to *bla*_NDM_ spread in livestock farms and in retail meat in China (Liu et al., 2019; Zhang et al., 2019b).

IncX3 plasmids have been identified as the primary vectors for the horizontal transfer of *bla*_NDM-5_ on farms (Ho et al., 2018; Williams et al., 2018) and our study further confirms this. IncX3 plasmids normally encode a type IV secretion system (pilX1-11) that allows the exchange of genetic material within bacteria. IncX3 plasmids are also highly conjugatable and stable and exert no fitness costs on their bacterial hosts (Zhai et al., 2020). This characteristic may explain why NDM-positive bacteria still have been found in these settings even though carbapenem use is prohibited on livestock farms. In addition to the IncX3 plasmids, IncFIB and IncFII were also highly represented. IncFIB plasmids have been frequently detected in *E. coli* and has already been described as an ESBL gene carrier (Zhang et al., 2019a). IncFII plasmids are important in ARGs spread and are also the primary *mcr*-1 vector for *E. coli* (Xavier et al., 2016; Sugawara et al., 2019). Therefore, the three types of plasmids discussed above may serve as an important vehicles for the spread of ARGs among animals and humans.

## Conclusion

In this study we identified 88 NDM-positive isolates from animal farms and their surrounding environments in China. Notably, this is a large-scale survey of CRE covering a large region of China over three consecutive years. Phylogenetic analysis indicated considerable diversity for this population of CRE isolates taken from livestock farms. WGS analysis further determined that *bla*_NDM-5_ coexisted with other ARGs and also demonstrated a great diversity in the plasmid population; the latter provides important epidemiological information for the global dissemination of *bla*_NDM-5_. These results indicated that continuous monitoring of NDM-positive *E. coli* in livestock farms and their surrounding environments is required to ensure public health safety.

## Data Availability Statement

The datasets presented in this study can be found in online repositories. The names of the repository/repositories and accession number(s) can be found at: “NCBI BioProject—PRJNA826982.”

## Ethics Statement

The Institutional Review Board of South China Agricultural University (SCAU-IRB) approved the samples and bacteria protocols. All animal faeces were sampled under authorization from Animal Research Committees of South China Agricultural University (SCAU-IACUC).

## Author Contributions

YY and Y-HL conceived of this study. R-SY, XK, YZ, and JL performed the experiments and collected the data. XK, R-SY, YZ, and Z-YQ analyzed and interpreted the data. XK drafted the manuscript. YY revised the manuscript. X-PL and JS coordinated the whole project. All authors contributed to the article and approved the submitted version.

## Funding

This work was supported by the National Natural Science Foundation of China (31730097), Local Innovative and Research Teams Project of Guangdong Pearl River Talents Program (2019BT02N054), the Program for Innovative Research Team in the Ministry of Education of China (IRT_17R39), the Innovation Team Project of Guangdong University (2019KCXTD001) and the 111 Project (D20008). R-SY was funded by GDAS’ Project of Science and Technology Development (2020GDASYL-20200103031).

## Conflict of Interest

The authors declare that the research was conducted in the absence of any commercial or financial relationships that could be construed as a potential conflict of interest.

## Publisher’s Note

All claims expressed in this article are solely those of the authors and do not necessarily represent those of their affiliated organizations, or those of the publisher, the editors and the reviewers. Any product that may be evaluated in this article, or claim that may be made by its manufacturer, is not guaranteed or endorsed by the publisher.

## Supplementary Material

The Supplementary Material for this article can be found online at: https://www.frontiersin.org/articles/10.3389/fmicb.2022.912260/full#supplementary-material

SUPPLEMENTARY FIGURE S1 PFGE analysis of 77 *bla*_NDM_-positive *Escherichia coli* isolates from food-producing animals. Triangles represent that the corresponding isolates sharing similar PFGE patterns.

## Footnotes

^1^ https://blast.ncbi.nlm.nih.gov/Blast.cgi

^2^ http://rast.nmpdr.org/

^3^ https://www-is.biotoul.fr/

^4^ http://www.genomicepidemiology.org/
