# Supplementary figures and images for: Molecular Epidemiology of New Delhi Metallo-β-Lactamase-Producing Escherichia coli in Food-Producing Animals in China
Source: Front Microbiol. 2022 Jul 1;13:912260. doi: 10.3389/fmicb.2022.912260 (PMC9284025; doi:10.3389/fmicb.2022.912260)

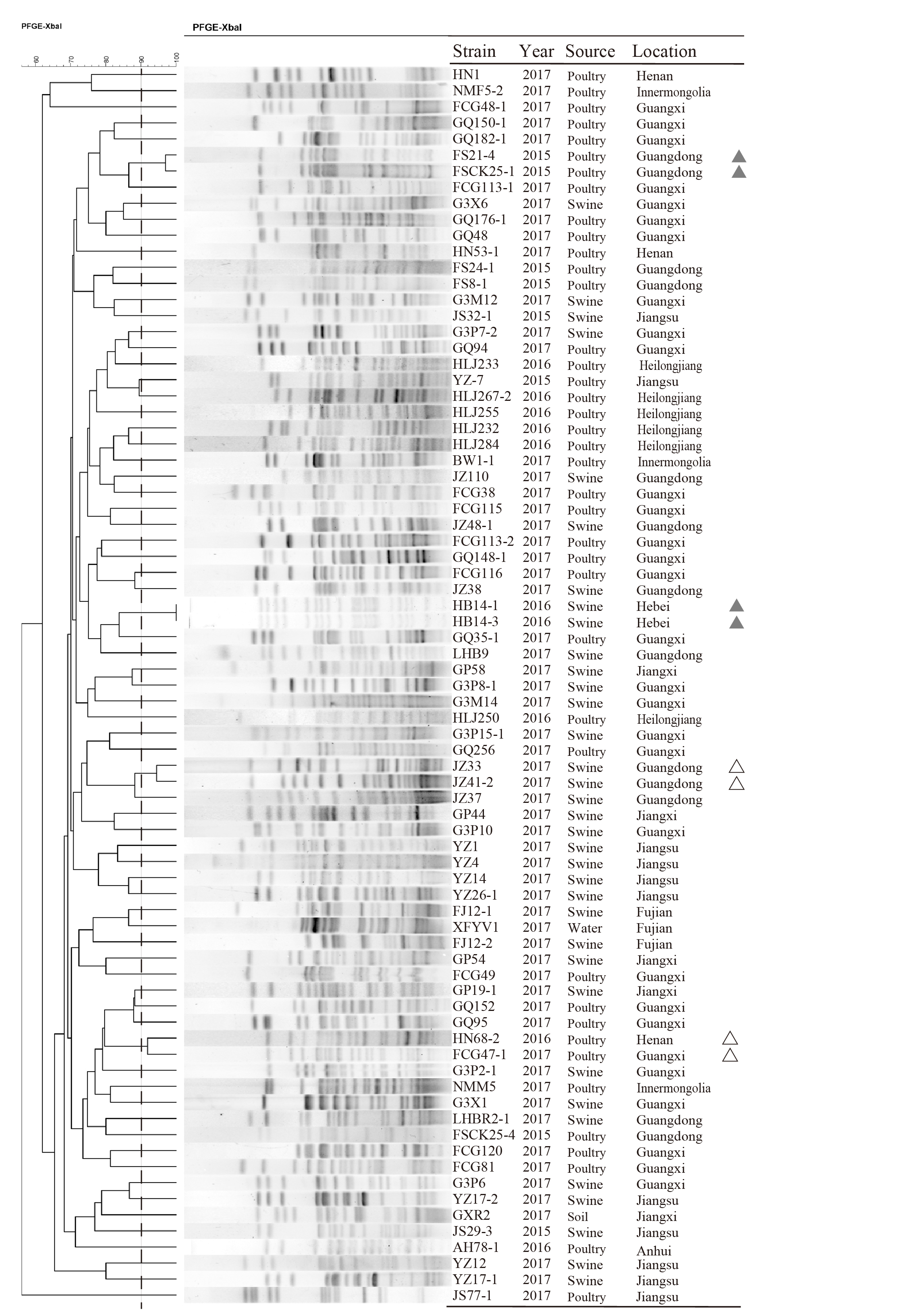

Supplement: SUPPLEMENTARY FIGURE S1 — PFGE analysis of 77 blaNDM-positive Escherichia coli isolates from food-producing animals. Triangles represent that the corresponding isolates sharing similar PFGE patterns. [file Figure_4.JPEG]
